# Conceptual transition from molecular to atomic: unleashing a new era in hydrogen therapy for chronic disease

**DOI:** 10.1093/nsr/nwae046

**Published:** 2024-02-21

**Authors:** Zhigang Zou

**Affiliations:** Eco-materials and Renewable Energy Research Center (ERERC), Jiangsu Key Laboratory for Nano Technology, National Laboratory of Solid State Microstructures and Department of Physics, Nanjing University, China

The dysregulated overexpression of reactive oxygen and nitrogen species (RONS) intricately links to the occurrence and development of diverse chronic diseases [1–3]. Hydrogen therapy stands out as a promising treatment methodology recognized for effectively mitigating RONS and maintaining intracellular redox equilibrium, thereby yielding therapeutic benefits [4,5]. In clinical settings, the prevalent non-invasive administration involves air, water, and saline solutions enriched with dissolved H_2_. However, the limited solubility of H_2_ poses challenges—its unguided transportation in the circulatory system risks depletion before reaching the target lesion, and its therapeutic efficacy remains stagnant at present. This dilemma prompts a critical inquiry: is molecular H_2_ the exclusive choice as a therapeutic medium?

Hydrogen, being the most abundant element in the cosmos, manifests in various natural forms that offer contemplations and alternations for the refinement of hydrogen therapy [6,7]. A recent publication in *National Science Review* by Zhu and colleagues marks a paradigm shift in this domain. Their research delves into the biological activity of hydrogen species in solid-state materials, unveiling an innovative therapeutic mechanism mediated by atomic hydrogen [8]. This pioneering approach offers greater controllability, precision and efficiency than conventional methods, thus avoiding many drawbacks of molecular H_2_ as the therapeutic medium through direct use of atomic hydrogen—a relatively stable form of hydrogen deposited in solid form that has longer *in vivo* lifespan, deeper tissue penetration, and exceptional broad-spectrum RONS scavenging capability.

First-principle calculations reveal the thermodynamic superiority of the highly reductive atomic hydrogen, favoring its direct reaction with malignant RONS to generate harmless water as the end product [9]. As a demonstration of this principle, the authors confirmed that tungsten-bronze phase H_0.53_WO_3_, an ideal carrier for atomic hydrogen, exhibits characteristics of high-content hydrogen storage, temperature-dependent hydrogen

release, and pH-responsive biodegradation, which allows for targeted accumulation and release as needed, which is unachievable with traditional molecular H_2_ as the therapeutic medium.

**Figure 1.**
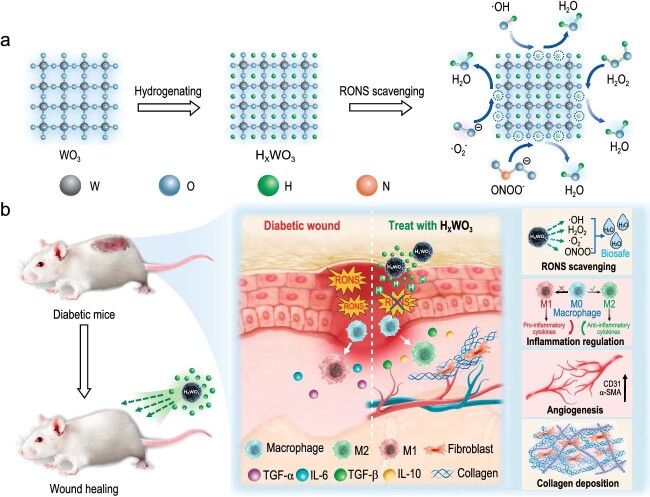
Schematic illustration of (a) solid-state atomic hydrogen as a strong and broad-spectrum RONS scavenger and (b) its therapeutic mechanism in diabetic wound healing. Reprinted from Ref. [8].

In chronic diabetic wound models, inflammation and ulceration caused by the excessive accumulation of RONS constitute the primary complications. Atomic hydrogen, through its exceptional RONS scavenging ability, effectively restores the diabetic wound microenvironment. This pivotal intervention leads to the regulation of beneficial anti-inflammatory cytokine expression, and thereby promotes cellular proliferation, collagen deposition and angiogenesis to effectively accelerate chronic wound healing (Fig. 1).

In summary, this research article by Zhu *et al*. represents an advanced therapeutic platform centered on atomic hydrogen, which significantly expands the basic research scope of hydrogen therapeutic biomaterials. Further, the innovative spirit epitomized by exploring hydrogen in different physical forms as efficient RONS scavengers provides valuable insights for future advancements in hydrogen therapy and its application in modern medicine.
